# Cascading time evolution of dissipative structures leading to unique crystalline textures

**DOI:** 10.1107/S205225251402288X

**Published:** 2015-01-01

**Authors:** Takeji Hashimoto, Hiroki Murase

**Affiliations:** a Department of Polymer Chemistry, Graduate School of Engineering, Kyoto University, Katsura, Nishikyo-ku, Kyoto, 615-8510, Japan; b Quantum Beam Science Directorate, Japan Atomic Energy Agency, Tokai-mura, Ibaraki, 1319-1195, Japan; c Professor Emeritus, Kyoto University, Kyoto, 606-8501, Japan

**Keywords:** dissipative structures, shish-kebab structures, extended-chain crystal textures, polymer crystallization

## Abstract

This article reports unique pattern formation processes and mechanisms *via* crystallization of materials under external flow fields as one of the general problems of open nonequilibrium phenomena in statistical physics. The external fields effectively reduce step-by-step the exceedingly large free energy barriers associated with the reduction of the enormously large entropy necessary for crystallization into unique crystalline textures in the absence of the fields. The cascading reduction of the free energy barrier was discovered to be achieved as a consequence of a cascading evolution of a series of dissipative structures. Moreover, this cascading pattern evolution obeys the Ginzburg–Landau law. It first evolves a series of large-length-scale amorphous precursors driven by liquid–liquid phase separation under a relatively low bulk stress and then small-length-scale structures driven by a large local stress concentrated on the heterogeneous amorphous precursors, eventually leading to the formation of unique crystalline textures which cannot be developed free from the external fields. Here the multi-length-scale heterogeneous structures developed in the amorphous precursors play a dominant role in the triggering of the crystallization in the local regions subjected to a large stress concentration even under a relatively small applied bulk stress.

## Abstract 2

This article reports unique pattern formation processes and mechanisms *via* crystallization of materials under external fields as one of the general problems in statistical physics of open nonequilibrium phenomena. Specifically, the article clarifies for the first time the role of external fields on the crystallization of homogeneous solutions of flexible polymers having enormously large conformational entropy into unique shish-kebab and extended-chain crystal textures.

## 1. Introduction   

In this article, we report a unique pattern formation process *via* crystallization in systems subjected to external flow fields. The systems to be discussed here have an extremely large entropy loss for crystallization and, thereby, the associated free energy barrier can hardly be overcome without the external fields. The external fields were discovered to effectively reduce the barrier step-by-step in a ‘cascading’ manner, enabling various ordered structures, which were initially amorphous, to form. We discovered these amorphous precursors provided excellent fields for the birth of unique crystalline textures even under a relatively low external bulk stress.

Systems open to an external flow of energy are brought into nonequilibrium and, thereby, the systems are called ‘open nonequilibrium systems’. The cascading pattern formation to be discussed hereinafter belongs to a problem in the general framework of the so-called open nonequilibrium phenomena in statistical physics. When the external fields in the form of shear flow, extensional flow, hot-drawing, *etc*., effectively reduce the entropy of the systems, the fields bring about ordered structures of the so-called ‘dissipative patterns’ (Nicolis & Prigogine, 1977 ▶). The various patterns formed in nature are dissipative patterns which vary with time at various relaxation times like cirrocumulus clouds in the sky caused by Rayleigh–Bénard convection or which are locked in *via* solidification like columnar joints in volcanic rocks.

**Figure 1:**
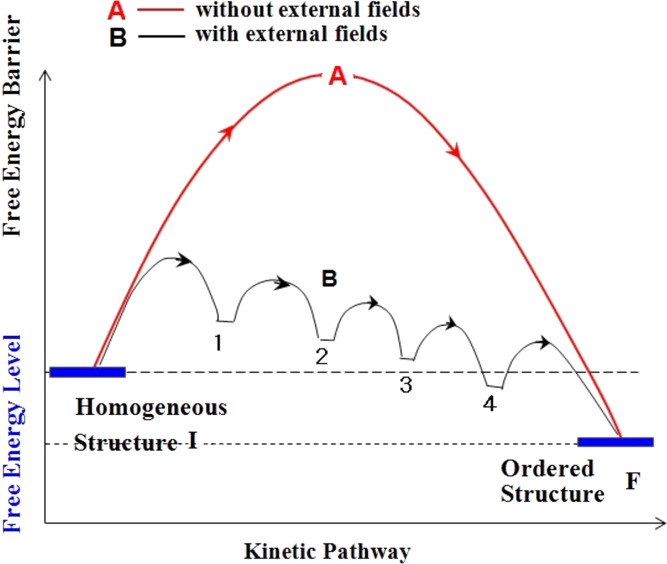
Concept of the flow-induced cascading reduction of a free energy barrier from A to B *via* cascading evolutions of a series of dissipative structures 1 to 4 for ordering from an initially homogeneous structure I into an ordered structure F. External fields assist to suppress an excessively large barrier A in the absence of the fields into a free energy landscape B, which enables the ordering from I to F that can be hardly attained in the absence of the fields.

The self-organization of molecular assemblies in open nonequilibrium binary solutions depends on a competition of two characteristic rates: the rate intrinsic to the solutions, Γ, and the rate externally imposed on the system. The intrinsic rate Γ = *Γ*(*q*) can be the relaxation rate of the local concentration fluctuations (CF) which generally depends on *q*, the wavenumber of the Fourier modes of the fluctuations (Landau & Lifschitz, 1964 ▶; Cahn & Hilliard, 1958 ▶; Doi & Onuki, 1992 ▶; Toyoda *et al.*, 2001 ▶). Frequently used abbreviations used in this work are listed in Table 1 ▶. The external rate can be the shear rate 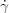 in the case of the simple shear flow being applied. If 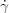 < Γ, the flow cannot affect the fluctuations, because the fluctuations are quickly decayed during the application of the flow. Thus, the shear flow would not essentially change the solution state: if it is homogeneous without the fields, it stays homogeneous even under the flow. However, if 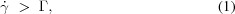
the fluctuations exist and hence are deformed during the application of the flow; thereby the flow changes the system state into a higher energy level. The flow-induced deformation may further enhance the building up of CF against osmotic pressure in some systems, as will be clarified later in §4.1. This building up of the CF is an effective energy dissipation mechanism for the solution under the flow into a lower energy level and creates the first flow-induced dissipative structure. If 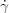 > Γ_d_, the relaxation rate of the first dissipative structure, the flow may further create the second dissipative structure starting from the first one, and so forth, leading to the cascading evolution of a series of dissipative structures under the flow, as schematically illustrated in Fig. 1 ▶.

Let us consider an ordering into a given ordered structure F from an initially homogeneous structure I under the conditions as shown in Fig. 1 ▶, where the ordering is thermodynamically favoured. Even under this condition, the ordering would never occur in the absence of the field in the case when the corresponding free energy barrier A is exceedingly high, as illustrated in Fig. 1 ▶. However, the applied field may lower the free energy barrier as illustrated in curve B *via* the cascading evolution of a series of the dissipative structures 1 to 4 for example; the evolution of each structure involves a small free energy barrier as illustrated by the anticipated free energy landscape B, which may lead to a novel and rational kinetic pathway for the ordering.

Consequently, the external fields may create a variety of dissipative structures which cannot be ever developed without the applied fields. The structures evolved may be arrested *via* vitrification and/or solidification (crystallization) processes, which open up a new method to manipulate nonequilibrium structures giving rise to novel properties or functionalities in advanced materials. In this work, we aim to explore basic principles underlying in the open nonequilibrium phenomena, specifically for a model system of a homogeneous solution of ultrahigh molecular weight flexible polymers.

## 2. Background   

### 2.1. Crystallization of homogeneous solutions into shish-kebab crystalline texture and extended-chain crystal texture   

**Figure 2:**
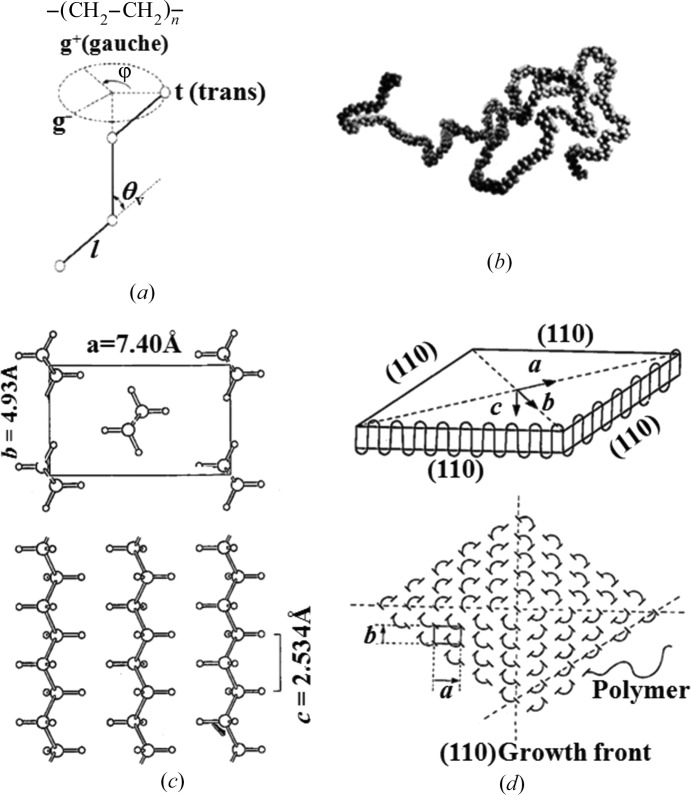
(*a*) Rotational freedom of chemical bonds under the fixed valence angle θ_v_, (*b*) a snapshot of the chain configuration in the amorphous phase, (*c*) chains in the crystal lattice and (*d*) the chain-folded lamellar crystal in polyethylene used for this study.

As a model flexible polymer, we used polyethylene (PE) which has the simplest chemical structure unit as shown on the top of Fig. 2 ▶(*a*). Each chemical bond has a rotational freedom with the potential minima at the rotational angle ϕ = 0° and 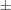
120°, designated, respectively, as *t* (*trans*), *g* ^+^ and *g* ^−^ (*gauche*) as shown in Fig. 2 ▶(*a*). If an ultrahigh molecular weight PE (UHMWPE) has a molecular weight of 2.0 × 10^6^, its number of bonds ∼1.4 × 10^5^ 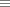 140 K. If each bond adopts the three rotational states (*t*, *g* ^+^, *g* ^−^), the number of possible states per chain Ω is ∼3^140K^. Thus, each chain has an extremely large conformational entropy; the chain has a random coil conformation in amorphous melts and solutions as shown in Fig. 2 ▶(*b*) (Flory, 1967 ▶). The probability of having a fully extended chain conformation with all bonds being *t* (*trans*) ∼ (1/3)^140K^ ∼ 0.

When the chains are crystallized, they are incorporated in the crystal lattice composed of an orthorhombic unit cell with all bonds in a *trans* conformation as shown in Fig. 2 ▶(*c*). At the limit where all chains are stretched out and packed in the orthorhombic crystal lattice, we could attain the extended chain crystal (ECC) with its length parallel to the *c* axis ≃ 20 µm for the given molecular weight. However, the ECC has never been obtained by crystallization from homogeneous solutions composed of random coils without applied fields because of an infinitely large conformational entropy loss and, thereby, an infinitely large free energy barrier encountered by the crystallization process. The most fundamental crystalline texture found for flexible polymers is the chain-folded lamella (Keller, 1958 ▶; Geil, 1963 ▶) as shown in Fig. 2 ▶(*d*) as a consequence of a balance of the two opposing physical factors encountered by the crystallization process: the cost of conformational entropy loss and the gain of bulk energy reduction. In this article, we define the ECC as extended-chain crystal ‘texture’, because it may contain defects such as chain ends, chain-folding parts, *etc*.

**Figure 3:**
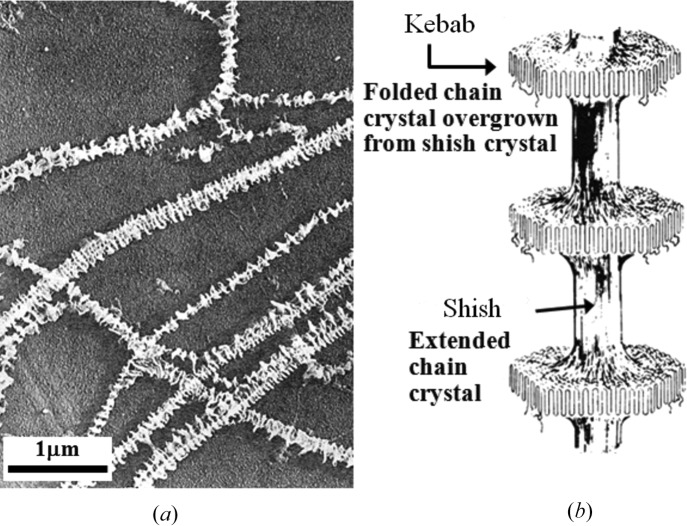
(*a*) TEM image of the shish-kebab crystalline texture [reproduced from Pennings *et al.* (1970 ▶)] and (*b*) its model.

About a half century ago, the shish-kebab crystalline texture (SK) was discovered in stirred polymer solutions (Mitsuhashi, 1964 ▶; Pennings & Kiel, 1965 ▶). Fig. 3 ▶ presents the transmission electron micrograph (3*a*) ▶ reported by Pennings *et al.* (1970 ▶) and its model (3*b*) ▶. The SK is a most intriguing but fundamental crystalline texture in polymers, as it has a mixed feature of ECC and chain-folded lamellae. It is composed of the so-called ‘shish’ crystal as a central core and the so-called ‘kebab’ crystal. The shish crystal is the ECC, while the kebab crystal is the chain-folded lamella epitaxically overgrown on the surface of as-grown shishs from random coils coexisting with the shishs. Since the discovery of the SK, a great number of researchers have been trying to clarify its formation mechanism and kinetic pathway. Nevertheless, to date the mechanism and pathway are not yet fully understood [see review articles by Keller & Kolnaar (1997 ▶) and Somani *et al.* (2005 ▶)].

It is most important to understand in depth the mechanisms which enable the reduction of the extremely large conformational entropy of macromolecules for the crystallization into the ECC and the SK. This is because they are composed, respectively, of essentially fully extended chains and a significant (or substantial) amount of fully extended chains. How do the applied fields assist the crystallization into the SK and the ECC, through cascading formation of what kind of dissipative structures and through what kind of mechanisms? We clarified these fundamental questions based on online, *in situ* explorations of time evolutions of the dissipative structures with a rheo-optical method (Hashimoto & Noda, 2012 ▶) involving simultaneous measurements of stress (σ), birefringence (Δ*n*), optical microscope (OM) images, and small-angle light scattering (SALS). Fine details of the dissipative structures captured by the online investigation were further explored by offline *ex situ* observations with transmission electron microscopy (TEM).

### 2.2. Self-assembly in dynamic asymmetric systems   

Our systems belong to the so-called ‘dynamically asymmetric systems’ in which components of the systems (polymer molecules and solvent molecules or colloidal particles and solvent molecules in the case of polymer solutions or colloidal dispersions, respectively) have a very different mobility. The self-assembly in the dynamically asymmetric systems has been relatively less explored than that in the dynamically symmetric systems, despite the fact that the systems may be more general and frequently found in nature.

Structure formation generally involves growth of spatial CFs which naturally involves a building up of local stress and stress relaxation. This generally occurs commonly in systems with or without the external fields. In the dynamically symmetric systems, this ‘intrinsic’ local stress is equally born by the components and relaxed at equal rates, usually faster than the growth rate of the CF in the course of the structure growth process; thereby giving rise to no explicit effects on the structure formation. However, in the dynamically asymmetric systems, this intrinsic local stress is primarily born by the smaller mobility (slower) component, because the faster component relaxes much faster than the counterpart. This local stress born by the slow component and the stress relaxation affect the free energy functional of the systems and hence cooperative diffusivity of the slower component, which is coined ‘stress-diffusion coupling’ (Doi & Onuki, 1992 ▶; Onuki, 2002 ▶). This stress-diffusion coupling should be reflected back to the pattern growth and to the form of the growing patterns (Tanaka, 1993 ▶; Toyoda *et al.*, 2001 ▶; Hashimoto, 2005 ▶).

It is believed that pattern formation in nature, including various materials and various systems of living bodies, may generally involve the stress-diffusion coupling effects. It is needless to say that the effects brought by the stress imbalance and the stress-diffusion coupling are important with regards to the pattern formation not only in the absence of the external fields but also in the presence of the fields (Helfand & Fredrickson, 1989 ▶; Milner, 1993 ▶; Onuki, 1997 ▶). Below we shall present the unique pattern formation processes for the system having extremely large dynamic asymmetry under the applied field.

## 3. Experimental systems   

We will test the concept proposed in the preceding sections by using the following two experimental systems, *i.e.* solutions of crystallizable and noncrystallizable ultrahigh molecular weight polymers. The solutions of crystallizable UHMWPE having weight-average molecular weight *M*_w_ = 2.0 × 10^6^ and weight-average degree of polymerization *n*_w_ 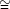 7.1 × 10^4^ are designated as Solution 1. The polymers in Solution 1 have an extremely large conformational entropy, thereby causing an infinitely large cost of entropy loss and an infinitely large free energy barrier, when they are crystallized into the ECC and the SK as described in §2.1. The solvent used is paraffin having a degree of polymerization of ~18 or decalin, so that the components comprising the solutions have an extremely large dynamic asymmetry. The solutions of a noncrystallizable ultrahigh molecular weight atactic polystyrene (UHMWaPS) having *M*_w_ = 5.48 × 10^6^ in dioctyl phthalate (DOP) were designated as Solution 2. Solution 2, also having the extremely large dynamic asymmetry, can evolve only amorphous dissipative structures due to liquid–liquid (L–L) phase separation, while Solution 1 can evolve not only the amorphous dissipative structures but also crystalline dissipative structures due to crystallization. Thus, comparisons of the dissipative structures evolved in these two systems provide important information concerning roles of phase separation and crystallization on the SK formation.

Solution 1 with paraffin as a solvent, at temperatures higher than its melting point *T*_m_ = 69°C, had the polymer concentration *C* = 5.0 wt% or *C*/C* = 10, where C* is the overlap concentration (de Gennes, 1979 ▶) and used for the online rheo-optical experiments to be described in §4.2.1. Solution 1 with decalin as a solvent had *C* = 10.0 wt% or *C*/C* = 20 and was used for the offline experiments to be described in §4.2.2. Solution 2 has the polymer concentration *C* = 6.0 wt% or *C*/C* = 40. These two solutions, Solution 1 and Solution 2, in which polymers are highly entangled, are elastic for a short time scale but viscous for a long time scale. They are thermodynamically stable, homogeneous solutions at our experimental temperatures in the absence of the applied fields, involving no crystallization and no phase separation. Thus, our systems are simple in a sense that the initially homogeneous systems will develop dissipative structures only after being subjected to the applied fields.

## 4. Results: flow-induced evolutions of dissipative structures into SK and ECC   

### 4.1. Shear-induced concentration fluctuations and phase separation   

When the homogeneous, single-phase solution is subjected to the simple shear flow with a shear rate 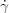 larger than a critical rate Γ_dis_, the disentanglement rate or the inverse of the longest rheological relaxation time (τ_m_), the solution was found to change from a transparent solution to a turbid solution and to bring about related changes in the rheological behaviour (Ver Strate & Philippoff, 1974 ▶). These intriguing and striking results suggest that some kinds of dissipative structures are formed under the given flow field as revealed by flow-induced SALS patterns (Hashimoto *et al.*, 1990 ▶; Hashimoto & Fujioka, 1991 ▶). From such observations as described above, it is important to note that the applied flow elevates the critical temperature, which brings about an initially single-phase solution into a phase-separating solution. It should be noted that this shear-induced L–L phase separation, caused by the stress-diffusion coupling inherent in the dynamically asymmetric systems, commonly occurs for solutions of both crystalline (Murase *et al.*, 2009 ▶; Hashimoto & Noda, 2012 ▶) and noncrystalline polymers (Hashimoto, 2005 ▶, 2008 ▶). We shall discuss the flow-induced CF and L–L phase separation on noncrystalline polymers below in this section and on crystalline polymers in §4.2.

**Figure 4:**
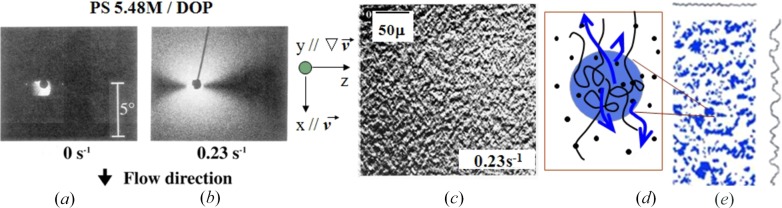
Shear-SALS patterns at (*a*) 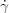 = 0 and (*b*) 0.23 s^−1^ and (*c*) shear-OM image for Solution 2. The model (*d*) schematically presents the ‘solvent-squeeze’, as illustrated by the blue arrows, from polymer-rich regions represented by the blue region to polymer-poor regions represented by the bright matrix to account for building up (*e*) the PLWCF with its wavevector parallel to the *x* axis. The dark dots in (*d*) schematically display some solvent molecules and the blue regions in (*e*) represent polymer-rich regions. The patterns on the right side and top side of (*e*) schematically represent the spatial CF along the *x* axis and *z* axis, respectively. The pattern (*e*) corresponds to that obtained by zooming out of the pattern (*d*). In (*a*) the bright part around the incident beam stop (a dark circle) is an artefact due to a portion of stray incident beam. The snapshots (*b*) and (*c*) were taken with shutter speeds of 10^−3^ and 10^−4^ seconds per frame, respectively.

Fig. 4 ▶ presents typical experimental results, on SALS patterns (shear-SALS; Figs. 4*a* and 4*b*) and an OM image (shear-OM: Fig. 4 ▶ *c*), observed on the shear plane (the *x*–*z* plane) with an incident beam propagating parallel to the velocity gradient (∇**v**) direction (*y* axis, normal to the plane of the paper) for Solution 2. All the data to be presented hereafter were taken under this condition. The results were obtained by using the shear rheo-optical method with a cone-and-plate fixture made out of quartz as detailed elsewhere (Hashimoto & Kume, 1992 ▶; Moses *et al.*, 1994 ▶; Matsuzaka & Hashimoto, 1999 ▶; Saito *et al.*, 1999 ▶; Hashimoto, 2005 ▶). The solution at rest (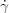 = 0 s^−1^, Fig. 4 ▶ *a*) is in a single-phase state, so that it does not exhibit an appreciable SALS and no features in the OM image. Upon imposing a flow of 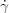 = 0.23 s^−1^ (Fig. 4 ▶ *b*), the strong SALS appeared along the flow direction (FD; the *x* axis) but scattering along the neutral direction (ND; the *z* axis) remained almost unchanged from that for the quiescent solution, so that the shear-SALS pattern exhibits the so-called ‘butterfly pattern’ having its bright wings along the *x* axis, while keeping the same darkness as the pattern in Fig. 4 ▶(*a*) along the *z* axis (defined hereafter as dark sector). The OM image in Fig. 4 ▶(*c*) taken simultaneously with the pattern (Fig. 4 ▶ *b*) exhibited some anisotropic contrast variations with the characteristic length of the order of 10 µm along the *x* axis. The fast Fourier transformation of the OM image reproduced the shear-SALS pattern (Fig. 4 ▶ *b*), revealing that the shear-OM image reflects the shear-flow-induced dissipative structures in the solution. We explain below the basic physics underlying the patterns in Figs. 4( ▶ *b*) and 4( ▶ *c*).

Even in the thermodynamically stable solution in a single phase state, there are thermal CFs and thereby regions rich in polymers having more entanglements as schematically illustrated in the blue region in Fig. 4 ▶(*d*) and regions poor in polymers having a fewer entanglements [the bright matrix in Fig. 4(*d*)]. Under the applied field, the polymer-rich regions bear larger stress than the other regions, through the concentration dependence of viscosity η and the coefficient of the first normal stress difference Ψ_1_, giving rise to local stress variations. However the stress developed by the flow can be relaxed *via* disentanglements, when 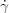 < Γ_dis_, so that the solution remains homogeneous even under the flow.

In the case when 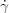 > Γ_dis_, deformed swollen entangled networks of polymers cannot be relaxed by the disentanglements. The elastic energy built up in the system by the deformation, however, can still be relaxed through squeezing solvents from the polymer-rich regions to the polymer-poor regions against osmotic pressure, as schematically illustrated by the blue arrows in Fig. 4 ▶(*d*) (Saito *et al.*, 1999 ▶). This process which enhances the CFs effectively dissipates the energy imposed on the solution to a lower energy level, because swollen deformed network chains can have relaxed conformations upon the solvent-squeezing process. Since the deformation of the chains are anisotropic, the solvent squeeze also occurs along the *x* axis, and hence the shear-enhanced CFs also become anisotropic as schematically illustrated in Fig. 4 ▶(*e*), where polymer-rich regions are shown in blue: the amplitude of the CF is large along the *x* axis, while that along the *z* axis is as small as the quiescent solution, giving rise to the plane-wave-type CF (PLWCF) with its wavevector **k** parallel to the *x* axis.

**Figure 5:**
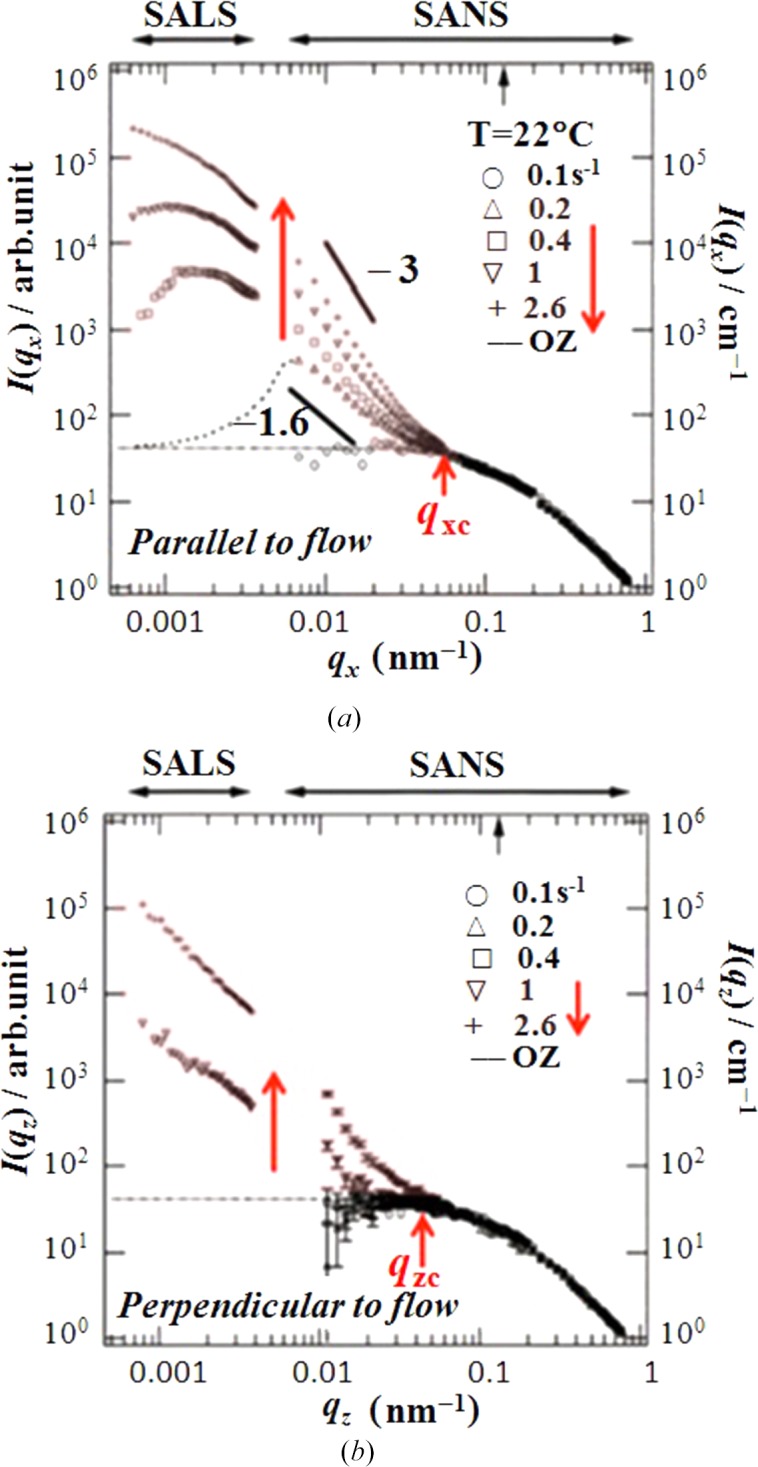
Steady-state scattering functions as a function of 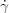 over a wide range of *q* [wavenumber of Fourier modes of the CF or magnitude of the scattering vector defined as *q* = (4π/λ) sin (θ/2) with λ and θ being the wavelength of the incident beam and scattering angle θ in the medium]. The small-angle neutron scattering (SANS) data were taken with the D11 spectrometer at ILL, Grenoble, France, with a set of sample-to-detector distances of 35.7, 10.0 and 2.5 m. The sample used was deuterated UHMWaPS having *M*_w_ = 2.0 × 10^6^ in DOP. *C* = 8.0 wt%, *C*/C* = 6.4 and *T* = 22°C much higher than the cloud point (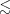
2°C). Here *q*_*x*_ and *q*_*z*_ are magnitude of the scattering vector (**q**) parallel to the *x* axis and *z* axis, respectively. The data were reproduced from Saito *et al.* (2002 ▶).

What about the 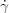-dependent dissipative structures developed over a wide length scale ranging from nm to µm under the steady shear flow? The fundamental question raised above can be answered in part by Fig. 5 ▶ which presents the shear-induced steady-state scattering functions *I*(*q*_*x*_, 0) and *I*(0, *q*_*z*_), proportional to structure factors *S*(*q*_*x*_, 0) and *S*(0, *q*_*z*_), parallel (Fig. 5 ▶ *a*) and perpendicular (Fig. 5 ▶ *b*), respectively, to the FD as a function of 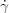 (Saito *et al.*, 2002 ▶). Note that the sample solution used here is slightly different from Solution 2 for the sake of convenience of SANS measurements. However, general behaviours to be described below are universally applicable to Solution 2 and even to Solution 1. At the low shear rate 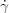 = 0.1 s^−1^ (Fig. 5 ▶ *a*) or 0.1, 0.2 and 0.4 s^−1^ (Fig. 5 ▶ *b*), the scattering functions were identical to that for the single-phase solution at rest (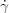 = 0 s^−1^) and are predicted by the Ornstein–Zernike equation
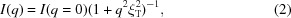
for thermal CFs in the solution at rest, as shown by the dotted lines which illustrate the crossover in the *q*_*K*_ dependence of the scattered intensity from 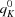 to 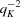 with increasing *q*_*K*_ across *q*_*Kc*_ (*K* = *x* or *z*). ξ_T_ is the thermal correlation length. Thus, we conclude that these shear rates are too small, satisfying the condition 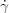 < Γ_dis_, so that the solution remains homogeneous under the flow, as discussed earlier in this section.

However, as 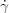 further increased, both the SALS and the SANS intensity were increased in both directions in the *q* range satisfying *q*_*x*_ < *q*_*xc*_ and *q*_*z*_ < *q*_*zc*_, where we found *q*_*xc*_ 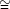 *q*_*zc*_ 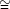 *q*_*c*_ ∼ 2π/ξ_T_, revealing that some dissipative structures having the length scale greater than ξ_T_ ∼ 2π/*q*_*c*_ ∼ 130 nm were developed; they grew with 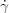. It is expected that the relaxation rate of the CFs in the *q* range satisfying *q*_*x*_ > *q*_*xc*_ and *q*_*z*_ > *q*_*zc*_, defined respectively as Γ (*q*_*x*_ > *q*_*xc*_) and Γ (*q*_*z*_ > *q*_*zc*_) are much higher than 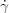 = 2.6 s^−1^, so that the applied shear at these rates cannot ever affect such high-*q* (or short length scale *l*) Fourier modes of the fluctuations as *q* > *q*_*c*_ and hence cannot build up the structural element having such small *l*. One needs much higher 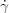 for this purpose. Generally, the relaxation rate Γ(*q*) increases with *q*, because the free energy *F*(*q*) for the *q*-Fourier mode of CF increases with *q* as follows in the context of the Ginzburg–Landau law (Landau & Lifschitz, 1964 ▶) and the Cahn–Hilliard law (Cahn & Hilliard, 1958 ▶),
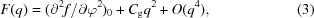


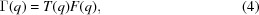
where *f* is the free energy density of polymer solutions with ϕ being the volume fraction of polymers, *C*_g_ is a positive *q*-independent constant related to the gradient free energy, and *T*(*q*) is the *q*-dependent transport coefficient. For a single phase solution, 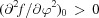. Therefore, the shear enhancement of the scattering intensity is expected to decrease with *q* as shown in the figure. It is important to note that the structural elements with small-*q* values or large length *l* are more enhanced by the flow. Thus, we focused on shear-enhanced SALS in the experiment on Solution 1 to be discussed in §4.2.1.

At a given 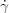, we found generally that *I*(*q*_*x*_) > *I*(*q*_*z*_), elucidating that the shear-induced dissipative structures have an anisotropy with respect to the shear direction as will be detailed in the next section. Moreover, in the shear-rate range 0.2 < 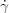 < 0.4, only *I*(*q*_*x*_) is enhanced by shear, but *I* (*q*_*z*_) still remains unaffected by shear, suggesting that the shear created the PLWCF with its **k** parallel to the FD, as discussed earlier in §4.1 in conjunction with Fig. 4 ▶. On the other hand at 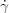 > 1 s^−1^, the scattering intensity *I*(*q*_*z*_) along the ND also is enhanced, which is a nonlinear effect indicative of creation of the interface along the ND also. This implies a transformation of the dissipative structure from the PLWCF into the small phase-separated domains with increasing 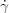, though their interfaces may be still rough (Saito *et al.*, 2003 ▶).

### 4.2. Flow-induced evolutions of dissipative structures into SK   

#### 4.2.1. Online rheo-optical investigations   

**Figure 6:**
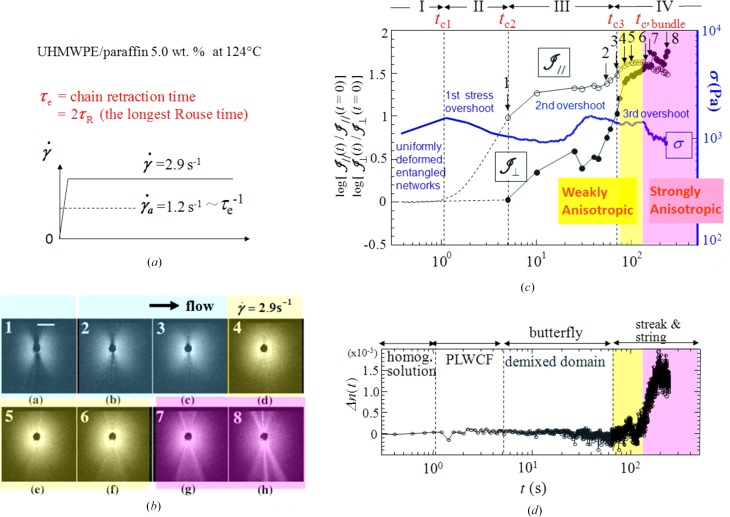
Time evolution of simultaneously measured SALS patterns (*b*), the integrated SALS intensities parallel (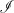
_//_) and perpendicular to the FD (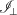), σ(c), and the birefringence Δ*n*(*t*) (*d*) after a step-up 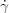 from 0 to 2.9 s^−1^ (*a*). 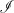
_//_ and 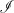
_⊥_(*t*) were normalized with 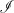
_//_ and 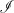
_⊥_(*t*) at 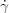 = 0 s^−1^, respectively. Δ*n* 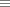 *n*_*z*_ − *n*_*x*_, where *n_k_* (*k* = *x* or *z*) is the refractive index along the *k* axis. The data based on those reported by Hashimoto & Noda (2012 ▶).

**Figure 7:**
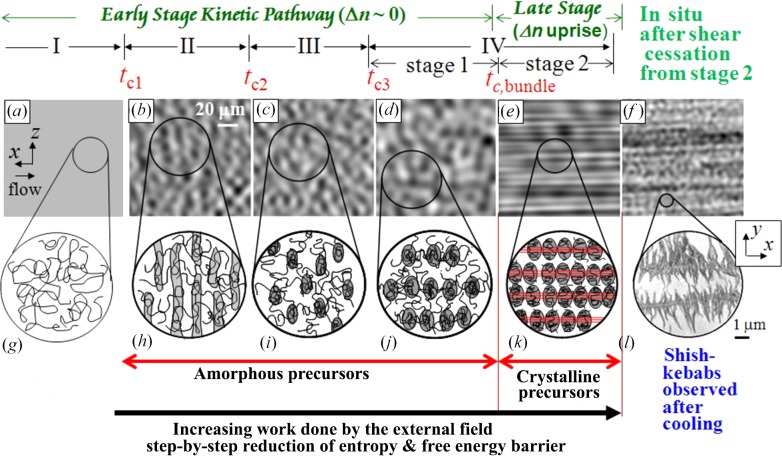
Online snapshots of the OM images (*a*) to (*f*) simultaneously measured with the results shown in Fig. 6 ▶ and their corresponding sketches (*g*) to (*k*) and a TEM image (*l*). Each snapshot was taken at a shutter speed of 10^−4^ s. The time regions I to IV are common with those defined in Fig. 6 ▶(*c*).

We explored time evolution of a series of the dissipative structures at real time and *in situ* for the UHMWPE solution (Solution 1) described in §3 after a step up for 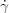 from 0 to 2.9 s^−1^, higher than the critical shear rate 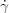
_a_ ∼ τ_e_^−1^, by the shear rheo-optical method (Hashimoto *et al.*, 2010 ▶). Here τ_e_ is the chain retraction time required for the stretched chain being relaxed with the Rouse mode (as shown in Fig. 6 ▶ *a*). Fig. 6 ▶ summarizes online snapshots showing the time evolution of the shear-SALS patterns 1–8 shown in Fig. 6 ▶(*b*) taken at particular times as indicated by the arrows together with the numbers in Fig. 6 ▶(*c*), the time evolution of the shear stress σ(*t*) and the integrated SALS intensity parallel and perpendicular to the shear flow 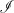
_//_(*t*)/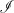
_//_(*t* = 0) and 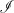
_⊥_(*t*) /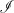
_⊥_(*t* = 0), respectively, normalized with that at *t* = 0 (Fig. 6 ▶ *c*), and the time evolution of the Δ*n* (Fig. 6 ▶ *d*), while Fig. 7 ▶ summarizes the online snapshots of the shear-OM images 7(*a*) to 7(*f*) together with the corresponding sketches 7(*g*) to 7(*k*) and the TEM image 7(*l*) obtained after cooling the structure shown in 7(*f*). All the results shown in Figs. 6 ▶ and 7 ▶ were simultaneously measured.

All those results shown in Figs. 6 ▶ and 7 ▶ indicate that the entire time-evolution process, covered up to ∼300 s in this particular experimental conditions, can be classified into four fundamental processes occurring in the time regions I–IV as specified in the figures. The results reveal the cascading time evolution of the dissipative structures. We shall summarize physical significance found in each region below.

(*a*) *Region I, *t* < *t*_c1_: incubation period*. Here *t*_c1_ defines the ‘solvent-squeezing time’. In this region, the OM image is featureless, not showing any distinct patterns, as schematically shown in Fig. 7 ▶(*a*), and the SALS pattern could not be discerned as revealed by 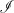
_//_(*t*) = 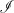
_⊥_(*t*) = 0 in Fig. 6 ▶(*c*), respectively. These results reveal that the sheared solution remained homogeneous; the entangled chains were more or less uniformly deformed as sketched in Fig. 7 ▶(*g*), thereby causing a stress uprise up to *t* ∼ 1 s as shown in Fig. 6 ▶(*c*).

(*b*) *Region II, *t*_c1_ < *t* < *t*_c2_: PLWCF*. Here *t*_c2_ defines the characteristic time for the formation of the demixed domains. In this region 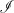
_⊥_(*t*) = 0, but 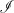
_//_(*t*) increased with *t* as shown in Fig. 6 ▶(*c*), suggesting the evolution of the butterfly-type SALS pattern 1 in Fig. 6 ▶(*b*), as a consequence of the building up of the PLWCF along the FD due to the solvent squeeze as found for Solution 2 and discussed in §4.1. This conclusion was confirmed by the real-space OM image shown in Fig. 7 ▶(*b*) and its sketch in Fig. 7 ▶(*h*), where the dark and the shaded regions represent those rich in polymers; the bright and the unshaded regions represent those poor in polymers. The conclusion is supported also by the first stress overshoot shown in Fig. 6 ▶(*c*), because the solvent squeeze causes the stress relaxation into a lower stress level as discussed in §4.1.

(*c*) *Region III, *t*_c2_ < *t* < *t*_c3_: demixed domains*. Here *t*_c3_ defines the characteristic time for the string formation. In this region, 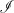
_⊥_ started to increase with *t* and hence the ‘dark sector’ of the butterfly pattern observed along the ND became less clear with *t* as shown by SALS patterns 2 and 3 in Fig. 6 ▶(*b*). The increase in 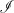
_⊥_ is a nonlinear effect driven by the flow and can be interpreted as a signature of a transformation of the PLWCF to demixed domains as discussed in §4.1. The physical origin of this transformation is considered as follows. As time elapses in this region, the amplitude of the PLWCF increases as a consequence of a progressive solvent-squeeze process, which causes (i) the PLWCF to be unstable, owing to the increasing hydrodynamic interactions between solvent flow and the PLWCF, as supported by (ii) the stress uprise at *t* > 20 s. Thus, the PLWCF will be eventually broken into the demixed domains, which (iii) creates the domain interfaces along the ND also, hence increasing 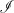
_⊥_; and (iv) causes the stress to decay, hence giving rise to the second stress overshoot centring around *t* ≃ 40 s. The OM image in Fig. 7 ▶(*c*) suggests formation of the demixed domains, whose mass centres are randomly placed in space as sketched in Fig. 7 ▶(*i*), consistently with the SALS and stress behaviours.

(*d*) *Stage 1 in Region IV, *t*_c3_ < *t* < *t*_c,bundle_: string with weak optical anisotropy*. Here *t*_c,bundle_ defines the critical time for formation of bundles of oriented chains (BOC) interconnecting the domains within the strings, as will be clarified below. In region IV, the demixed domains were aligned into string-like assemblies oriented parallel to the FD (the *x* axis) driven by hydrodynamic interactions between the domains and solvent flow, so that the centres of the domains within the strings were nematically aligned parallel to the FD, as shown in the OM images in Figs. 7(*d*) and 7(*e*) and their corresponding sketches (Fig. 7 ▶ *j*) and (Fig. 7 ▶ *k*). The strings can be recognized by the appearance of the streak-like scattering pattern oriented along the ND (the *z* axis) as shown in the patterns 4 to 8 in Fig. 6 ▶(*b*) and by the large intensity 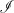
_⊥_ in region IV, while the demixed domains inside the string can be discerned by 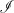
_//_ owing to the diffuse scattering along the FD. The physical significances of the multiple streaks in patterns 6 to 8 in Fig. 6 ▶(*b*) are discussed elsewhere (Murase *et al.*, 2009 ▶). It is striking to note that the Δ*n* dramatically increased at *t* ∼ *t*_c,bundle_ ∼ 125 s, the broader of the two regions marked in yellow and pink in Fig. 6 ▶. We define these two regions in region IV as stage 1 and stage 2, respectively.

(*e*) *Stage 2 in Region IV, *t* > *t*_c,bundle_: string with strong optical anisotropy*. The sharp crossover from the weak to the strong optically anisotropic strings at *t*_c,bundle_ is the most intriguing and fundamental physical process to be studied further in depth, because it must imply a coupling between the L–L phase separation and the liquid–solid phase transition (crystallization) at this particular time. In stage 2, 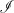
_⊥_ finally became larger than 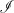//, indicating that the streak scattering due to the string as a whole becomes stronger than the diffuse scattering due to the domains aligned inside the strings, implying an increased number density of the domains within the strings. This enrichment of the domain density may trigger the formation of the BOCs interconnecting the domains within the strings as suggested by the Δ*n* uprise and by the strings having large optical anisotropy in Fig. 7 ▶(*e*). The corresponding sketch (Fig. 7 ▶ *k*) schematically illustrates formation of the BOCs in the strings by the red lines along the FD. The bundle formation must be intricately related to the third stress overshoot centring around *t* ∼ *t*_c,bundle_ ∼ 125 s as shown in Fig. 6 ▶(*c*). It is important to note that the BOCs may be formed *via* coil-to-stretched-chain transition (CSCT) localized within the strings rather than the CSCT induced in homogeneous solutions (de Gennes, 1974 ▶; Keller & Kolnaar, 1997 ▶; Dukovski & Muthukumar, 2003 ▶).

(*f*) *Summary on online rheo-optical studies*. In addition to the important pieces of information concerning the cascading pattern formation extracted from each of the time regions (II) to (IV) as described above, we shall further summarize below important physical factors discovered from the above experiments.

(i) The early stage kinetic pathway (up to stage 1 in region IV) evolves a series of the amorphous dissipative structures as shown in Figs. 7 ▶(*h*) to 7(*j*), which are amorphous precursors leading to the formation of the SK, because Δ*n* ∼ 0.
(ii) If the shear is stopped at any time regions in the pathway described in (i), the structures disappear to form the homogeneous solutions.
(iii) The later stage kinetic pathway, corresponding to stage 2 in region IV, is anticipated to involve the crystallization into shish crystals *via* nucleation from the BOCs and hence formation of crystalline precursors, as supported by the following pieces of evidence.
(iv) Strikingly, the OM image exhibiting the optical anisotropy (Fig. 7 ▶ *e*) did not disappear even after the shear cessation at 124°C as shown in Fig. 7 ▶(*f*), the intensity of the streak-like SALS pattern further increased with *t* (Murase *et al.*, 2005 ▶, 2009 ▶, 2011 ▶), and the solution after cooling showed the shish-kebabs under TEM, as shown in Fig. 7 ▶(*l*).
(v) Those results described in (iv) suggest that the crystallization into shishs occurred *in situ* under the flow at *t* > *t*_c,bundle_; this crystallization not only prevents relaxation of the structure created but also promotes further crystallization *in situ* at 124°C into shish-kebabs after the shear cessation, according to the mechanism proposed by Peterman and co-workers (Lieberwirth *et al.*, 2000 ▶), though the shish-kebabs shown in Fig. 7 ▶(*l*) might also grow during the cooling process of the solution for the TEM observations.
(vi) Strikingly enough, the evolution of the series of the dissipative structures found for the UHMWPE solution (Solution 1) was the same as that found for the UHMWaPS solution (Solution 2) (Kume *et al.*, 1997 ▶; Murase *et al.*, 2009 ▶; Hashimoto & Noda, 2012 ▶), except for the fact that the optically anisotropic strings developed in stage 2 for the amorphous UHMWaPS could not be crystallized *in situ*. Thus, the BOCs of the UHMWaPS were relaxed back into the homogeneous solution, unless the solution is rapidly vitrified below its glass transition temperature. In §4.1, we explicitly presented a part of the dissipative structures, *i.e.* PLWCF and the demixed domains, evolved for Solution 2.
(vii) *General conclusion 1*. The work done to the solution by the flow, *W*, increases with *t*, which step-by-step reduces the entropy and free energy barriers *via* cascading time evolution of the amorphous precursors driven by the flow-induced L–L phase separation. The amorphous precursor in stage 1 provides a special template which efficiently promotes the crystallization into the SK.
(viii) *General conclusion 2*. The cascading pattern formation obeys the Ginzburg–Landau and Cahn–Hilliard laws given by equation (3) in a sense that Fourier modes of the structure elements having a smaller *q* and hence a larger *l* have a larger driving force for growth; the larger structures evolve first under a low average stress, σ_b_ ∼ *O*(10^3^ Pa), as shown in Fig. 6 ▶(*c*), driven by the L–L phase separation, followed by the evolution of the small structures driven by crystallization under a low σ_b_ but a high local stress σ_l_, as will be clarified in the next section. The amorphous precursors having the large structures with the characteristic length of the order of 20 µm cannot be observed by SAXS and SANS and also by conventional birefringence experiments as Δ*n* ∼ 0, so that their existence has been overlooked for a long time up to now since the discovery of the shish-kebabs.

#### 4.2.2. Offline TEM investigations   

**Figure 8:**
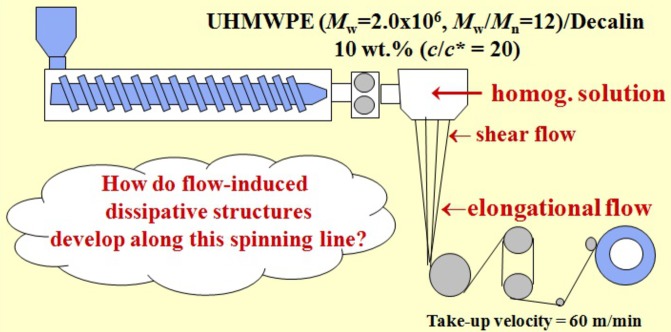
Schematic illustration of the fibre spinning experiment and some keywords in the spinning process.

In this section we shall investigate morphological details of a series of the dissipative structures with an aim of disclosing the secrets of the crystallization into the SK and to discover general principles of the cascading pattern formation in our systems, as one of the important themes in the open nonequilibrium phenomena.

(*a*) *Methodology*. Along with the aim described above, we set a challenge to investigate a series of the structures evolved as a function of positions along the fibre in a fibre spinning process with *ex situ*, offline TEM observations as detailed elsewhere (Murase *et al.*, 2011 ▶) and to be briefly described below. Fig. 8 ▶ schematically illustrates the fibre spinning process. The homogeneous solution of the UHMWPE in decalin (Solution 1) at 170°C was extruded through a spinneret; the extrudates were drawn into fibres which were cooled down by the nitrogen gas flow at room temperature. The fibres along the spinning line were first subjected to the shear flow and then to the elongational flow and/or deformation as indicated in the figure.

**Figure 9:**
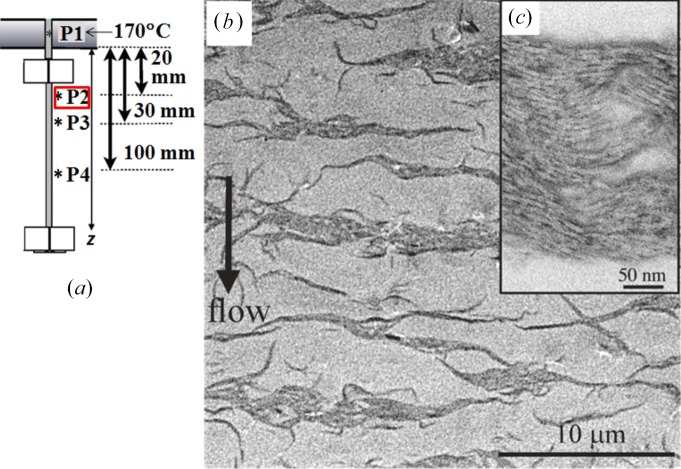
Typical TEM images (*b*) and (*c*) of the specimen at P2, 20 mm downstream from the nozzle as illustrated in (*a*). Image (*c*) is a zoomed-in image of the dark part in the image (*b*). The data based on those given by Murase *et al.* (2011 ▶).

The fibre running through the spinning line was quickly sampled out from the spinning line with a pair of frames having razor blades at its upper and lower edges to cut off and clamp the running fibres. The specimen fixed by the clamping frames was quickly cooled to room temperature with a nitrogen gas flow in order to lock in the structures evolved in the running fibre *via* solidification (crystallization). The solidified specimens were still gel-like with a concentration of solvent close to but less than 90 wt% at the positions from P2 to P4 and 40 wt% at P5: the positions P2 to P4 and P5 will be defined later in Figs. 9 ▶(*a*) and 13(*b*), respectively. In order to obtain specimens suitable for TEM observations without changing the structures developed in the solidified specimens, we used the following ‘fixation technique’. The solvent was stepwise replaced first from decalin to acetone and then from acetone to epoxy monomer, as detailed elsewhere (Murase *et al.*, 2011 ▶). The epoxy monomer was then cured into epoxy resin at 60°C for ∼8 h. This fixation process was confirmed to hardly affect the structures in the solidified fibres.

**Figure 10:**
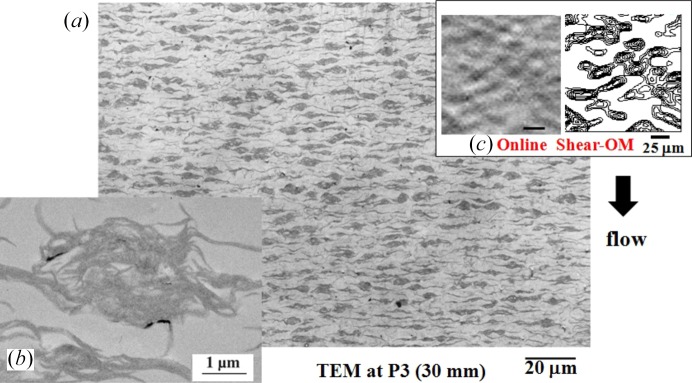
Typical TEM images (*a*) and (*b*) of the specimen at P3, 30 mm downstream from the nozzle, and (*c*) the corresponding online snapshot of the OM image under steady shear flow at 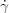 = 4.6 s^−1^ at 150°C for the same solution used in the experiments described in §4.2.1. The image (*b*) is a zoomed-in image of the dark domain in the image (*a*). The right-half of the OM image (*c*) shows a contour pattern composed of the iso-intensity lines of the left-half transmission OM image. The data are based on those given by Murase *et al.* (1995 ▶, 2011 ▶).

**Figure 11:**
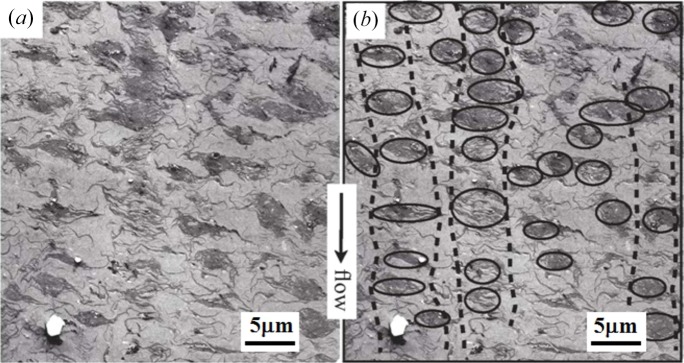
Typical TEM image of the specimen at P4, 100 mm downstream from the nozzle. Images (*a*) and (*b*) are the same except that (*b*) contains the outlines of the demixed domains (solid lines) and their string-like self-assembly (broken lines). The data are from Murase *et al.* (2011 ▶).

A block of the epoxy resin, in which the solidified specimen was embedded, was first trimmed to expose the inner region of the fibre for staining with RuO_4_ vapour for 10 h at room temperature. At room temperature, the stained specimen was then cut, with a diamond knife, parallel to the fibre axis close to the centre of the fibres into ultrathin sections of ∼70 nm thickness. The ultrathin sections used for the TEM observations had a length of ∼2 mm along the fibre axis and contained a whole fibre in the direction normal to the fibre axis. The RuO_4_ vapour seemed to condense selectively in the amorphous phase of PE to form fine nanoparticles which decorate the amorphous phase on the surfaces of the crystals developed by the solidification process. Thus, RuO_4_ staining gives a dark contrast to the PE phase and a bright contrast to the matrix phase of the solvent replaced by epoxy resin. Hereafter, we designate the matrix phase simply as the solvent phase for brevity. We conducted the offline TEM investigations of the dissipative structures evolved along the fibre spinning line as a function of the distance *z* from the nozzle, particularly at P1 to P4 as shown in Fig. 9 ▶(*a*) and P5 shown in Fig. 13(*b*) as the most interesting representative positions.

(*b*) *PLWCF at P2*. Fig. 9 ▶ shows a typical TEM image taken at P2, at *z* = 20 mm downstream from the nozzle as shown in Fig. 9(*a*). The dark PE-rich regions extended normal to the FD but their centres of mass arranged periodically along the FD were observed to be dispersed in the solvent matrix (Fig. 9 ▶ *b*). The dark region is composed of a stack of lamellar crystals with their lamellar normals oriented parallel to the FD (Fig. 9 ▶ *c*). The observed structure is believed to reflect well the online PLWCF with **k** parallel to the FD as sketched in Fig. 7 ▶(*h*), despite the observed structure being affected by the solidification process. Though the online structures at the small length scale are perturbed by the lamellar crystallization during the solidification process, the large-length-scale structures are well conserved, because their relaxation rates are much slower than the solidification rate. The fixation technique was found to be crucial in order to conserve a spatial distribution of the discrete structures dispersed in the solvent phase. A simple solvent evaporation method may not conserve the spatial distribution of the structures.

(*c*) *Randomly dispersed demixed domains at P3*. Fig. 10 ▶(*a*) shows a typical TEM image taken at P3, at *z* = 30 mm downstream from the nozzle. The image reveals that the dark polymer-rich domains had an oblate-ellipsoidal shape with the axis of revolution oriented parallel to the FD and their mass centres are randomly aligned in space. The domains themselves were solidified by the lamellar crystallization as shown in the inset (Fig. 10 ▶ *b*). Interestingly the offline image (Fig. 10 ▶ *a*) was quite similar to the online snapshot of the OM image shown in Fig. 10 ▶(*c*) (Murase *et al.*, 1995 ▶) in terms of both the size and the shape of individual domains and their spatial distribution. Thus the spatial distribution of the domains is believed to be well conserved in the offline TEM image through the fixation method. The shape of the domains and their orientation are believed to reflect a memory of the structure transformation process from the PLWCF to the demixed domains mediated by the hydrodynamic interactions as detailed in §4.2.1 (*c*).

(*d*) *Strings of demixed domains at P4*. Fig. 11 ▶ shows a typical TEM image taken at P4, at *z* ∼100 mm downstream from the nozzle. Figs. 11 ▶(*a*) and 11 ▶(*b*) are the same image, except that the domains and their alignments in space are outlined with the black line and the black dotted line, respectively, in Fig. 11 ▶(*b*). Those images clearly reveal such a trend that the demixed domains started to align with their centres of mass in rows parallel to the FD, as discussed in §4.2.1 (*d*), though there were still many off-aligned domains.

(*e*) *Coil-to-stretched-chain transition localized in bridge domains (BD) formed in dense strings at P4(+)*. The position P4 is a special position where the Δ*n* of the fibre sharply increased with a small increase of *z*, as it increased sharply with *t* across *t*_c,bundle_ in the online shear experiments as discussed in §4.2.1 (*e*). At a position slightly downstream from P4, defined as P4(+), the off-aligned demixed domains were extensively incorporated in the as-grown strings to enrich the number density of the domains within the given strings; such strings are defined hereafter as dense strings; moreover, the dense strings themselves tended to self-assemble into clusters, as shown in Fig. 9 ▶ in the paper by Murase *et al.* (2011 ▶). The enrichment of the domains within the strings and the self-assembling of dense strings into the clusters are driven by the hydrodynamic interactions with the solvent flow and the domains and those with the solvent flow and the strings, respectively. These phenomena are considered to reflect flow-induced macroscopic phase separation of the domains and strings against the solvent molecules (where the domains, strings, and solvent molecules comprise a kind of dynamic asymmetric system as discussed earlier in §2.2), resulting eventually in the syneresis of the solvent molecules from the fibre in the spinning line.

**Figure 12:**
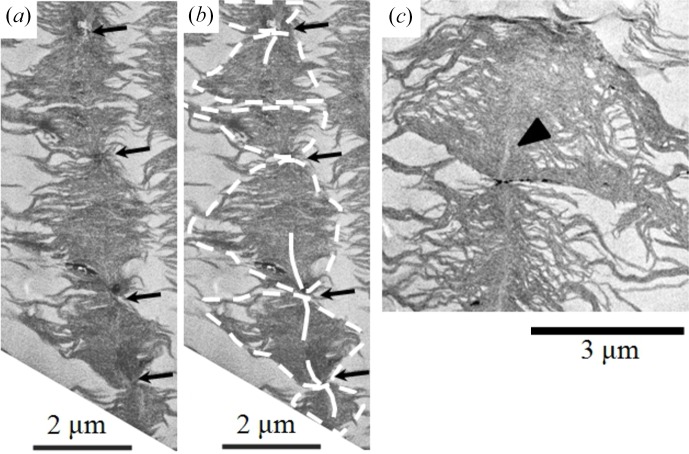
Typical TEM image of the specimen at P4 (+) which is only slightly downstream of P4. Images (*a*) and (*b*) are the same image except that (*b*) contains the outlines of the demixed domains in the string with the broken white lines and those of the shish crystals emanating from the interconnecting portions between the neighbouring domains with the white solid lines. View (*c*) highlights the shish crystals (bright line-shaped objects pointed out by the arrow) grown from the interconnected boundary of the two domains.

**Figure 13:**
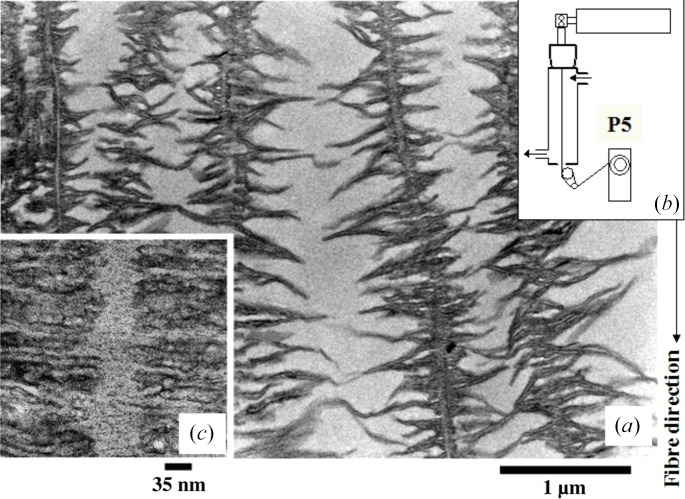
Typical TME image (*a*) of the specimen at P5 in the wound-up fibre (*b*) and the zoomed-in image of (*a*) highlighting the shish and overgrown kebabs (*c*).

The domains in the dense strings were finally interconnected with narrow and short bridge-like domains formed at their domain boundaries, defined hereafter as bridging domains (BDs), as highlighted by the dark arrows in Fig. 12 ▶. A close observation of the image (Fig. 12 ▶ *a*) revealed that bright line-shaped objects emanate from the BDs between the interconnected domains towards the centres of the domains, as outlined by the white lines emanating from the BDs toward the domain centres in Fig. 12 ▶(*b*). Fig. 12 ▶(*c*) shows a zoomed-in image of the two neighbouring domains interconnected by the BD, from which the bright lines emanate toward the centres of the domains. We anticipate that the bright lines are the shish crystals nucleated and grown from the BOCs, which in turn were developed *via* the localized CSCT within the BDs and were grown toward the centres of the domains. The lamellar crystals observed within the domains are expected to be grown during the cooling process of the specimen sampled from the spinning line. Growth of the BOCs in the BDs toward centres of the domains as well as nucleation and growth (defined hereafter as NG) of the shish crystals from the BOCs are believed to account for the sharp increase of Δ*n* with *z* across P4.

(*f*) *Shish-kebabs formed in as-spun fibres at P5*. Fig. 13 ▶(*a*) presents a typical TEM image at P5 in the wound-up as-spun fibres as sketched in Fig. 13 ▶(*b*). It shows well developed shish-kebabs swollen with solvent, the solvent concentration being decreased to ∼40 wt% due to the syneresis effect, which is discussed in §4.2.2 (*e*). The zoomed-in image in Fig. 13 ▶(*c*) clearly shows a well developed shish crystal in its centre and oriented along the fibre direction as well as well developed lamellar crystals overgrown laterally from the shish, both of which appeared to be bright, because the crystals were unstained by RuO_4_. The memory of the demixed domains is seen to significantly fade away due to long-range rearrangements of chains in crystals *via* the sliding chain diffusion (Hikosaka, 1987 ▶).

### 4.3. Hot-drawing-induced dissipative structures: pathways from SK to ECC   

How do hot-drawings, which are applied to the as-spun fibres as a kind of external field, transform their internal crystalline textures of the as-spun fibres from the SK into the ECC? This is also an intriguing open nonequilibrium phenomenon which gives rise to ultrahigh-strength PE fibres. The as-spun gel-like fibres were subjected to either a single-step drawing or a two-step drawing. The single-step drawing was conducted with a draw ratio ∊_s_ = 1.3, 2, 3 or 4 at *T* = 120°C close to the *T*_m_ of the as-spun fibre, while the two-step drawing was made as follows: the first-step drawing of the as-spun fibre with a draw ratio ∊_1_ = 2, 3 or 4 at temperature *T* = 120°C and the second-step drawing with a draw ratio ∊_2_ = 2, 3, 4, 5 or 6 at *T* = 145°C close to *T*_m_ for the fibre subjected to the first-step drawing.

**Figure 14:**
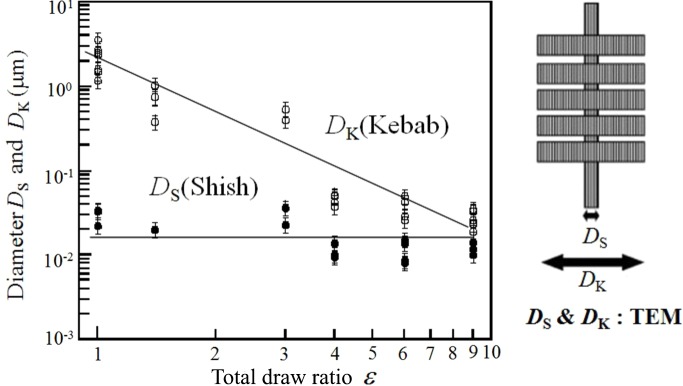
Average diameters of shishs and kebabs measured with a TEM as a function of total draw ratio ∊ attained by the single-step and/or the two-step hot-drawing of the as-spun fibre.

The fibres after the single-step or two-step hot-drawing were completely free from solvent. Some of the drawn fibres were picked up to investigate closely under a transmission electron microscope in order to evaluate the average diameters of shishs (*D*_S_) and kebabs (*D*_K_) using the RuO_4_ nanoparticles adhering on the surfaces of shishs and kebabs as useful markers. The results are plotted in Fig. 14 ▶ as a function of total draw ratios ∊, ∊ ≡ ∊_s_ for the single-step drawing or ∊ ≡ ∊_1_∊_2_ for the two-step drawing, in a double logarithmic scale. It is striking to note that *D*_S_ hardly changed with ∊, though *D*_K_ decreased with ∊ to the limiting value close to *D*_S_ at ∊ ∼ 9, which revealed that the drawn fibre at ∊ ∼ 9 is essentially composed of the shish crystals only and hence the ECC texture (Ohta *et al.*, 2010 ▶).

## 5. Discussion   

### 5.1. Hypothesis on cascading time evolutions of a series of dissipative structures into SK   

**Figure 15:**
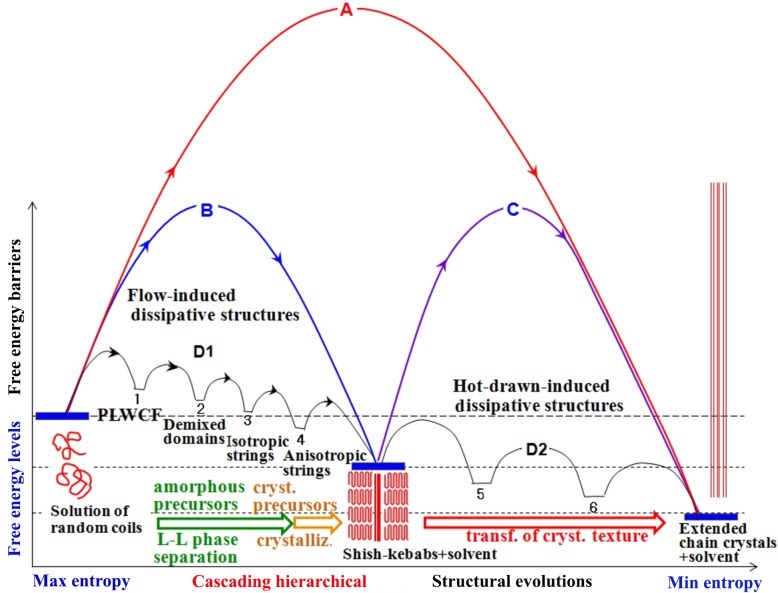
Cascading pattern formation from homogeneous polymer solutions to the SKs *via* the flow-induced formation of dissipative structures 1–4, following the free energy landscape D1, and cascading pattern formation from the SK to the ECCs *via* the hot-drawing-induced dissipative structures 5 and 6, following the free energy landscape D2. 1 is the PLWCF, 2 is the randomly arranged demixed domains, 3 is the optically isotropic strings, 4 is the optically anisotropic strings, 5 and 6 are the SK having a kebab fraction reduced stepwise and increased shish length. The barriers A and B illustrate the free energy barrier in the absence of the external fields from the homogeneous polymer solutions to ECC or to SK, respectively, while the barrier C illustrates that from SK to ECC. The barriers A to C are anticipated to be extremely large, because the corresponding patterns were never observed in the absence of the external fields.

On the basis of experimental results and the discussion presented in §4.2.1 and §4.2.2, we now propose a concept of the cascading time evolutions of a series of dissipative structures into SKs dispersed in solvent, starting from a homogeneous solution composed of entangled random coils of polymers swollen with solvent, as shown in Fig. 15 ▶. The exceedingly high free energy barrier B encountered by the crystallization of random-coil chains in the homogeneous solution into the SK in the absence of an applied field will be stepwise suppressed as shown by the free energy landscape D1 under the applied fields *via* the cascading evolution of a series of the dissipative structures 1–4 as experimentally clarified in §4.2.1 and §4.2.2. The structures 1–3 are the amorphous precursors for the SK which is driven by the flow-induced L–L phase separation inherent in the dynamically asymmetric systems, while structure 4 is the crystalline precursor for the SK driven by the special crystallization process already elucidated in §4.2.2 (*e*) and to be further discussed in §5.4. The hypothesis about the free energy landscape D1 has not been theoretically proven yet, and an exploration of it deserves further work.

It is needless to say that the free energy barrier A for the ECC being directly crystallized from the homogeneous solution without the applied fields may be much higher than the corresponding free energy barrier B for the crystallization process discussed above. Thus, it would be impossible to crystallize the ECC directly from homogeneous polymer solutions without the external fields.

### 5.2. Concept on cascading structure evolution from the SK to the ECC   

**Figure 16:**
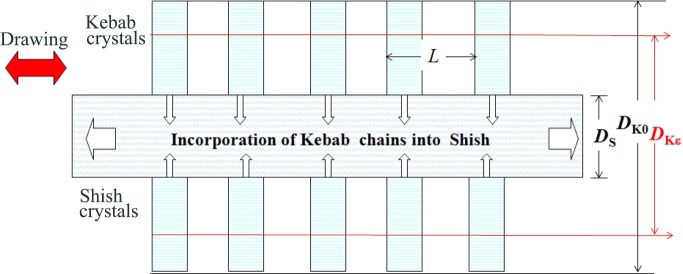
Hot-drawing-induced transformation of kebabs into shishs *via* the incorporation of chains in kebabs into shishs that induces decreasing *D*_K_ from *D*_K0_ to *D*_K∊_ with ∊ under almost constant *D*_S_ and *L*.

Based on the experimental results discussed in §4.3, we can also propose a concept about the cascading structure evolution from the SK to the ECC as shown in Fig. 15 ▶. The experimental results of decreasing *D*_K_ but almost constant *D*_S_ with ∊, as shown in Fig. 14 ▶, together with approximately constant lamellar spacing *L* during each step of the hot-drawing imply that the folded chains in the kebab crystals were incorporated into the shish crystals as schematically illustrated in Fig. 16 ▶. This hot-drawing process will result in decreasing *D*_K_ and fibre diameter, keeping constant *D*_S_ but increasing the length of fibre, shishs, and portions of bare shishs without kebabs, while essentially keeping a constant number of shishs *n*_S_. The number *n*_S_ may be primarily precontrolled by the number of the optically anisotropic strings formed as the amorphous precursor.

The exceedingly high free energy barrier C, as shown in Fig. 15 ▶, encountered by the transformation from the SK to the ECC without the applied field will be suppressed stepwise as also shown by the free energy landscape D2 under the multiple-step hot-drawings *via* the cascading evolution of the dissipative structures 5 and 6. The free energy landscape D2 in Fig. 15 ▶ is based on the three-step hot-drawing of the SK into the ECC. Each step of the hot-drawing gives the cascading structural change in the SK according to the mechanism, as schematically illustrated in Fig. 16 ▶, *via* sliding chain diffusions within the crystals as a primary mechanism, which may be responsible for the suppressed free energy barrier. Again, the hypothesis on the free energy landscape D2 has not been theoretically proven yet and is left for a future work. The experimental results for the given hot-drawing conditions revealed that
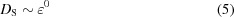
and 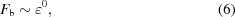
where *F*_b_ is the average force at break per single fibre (Ohta *et al.*, 2010 ▶). Equation (5) obtained from the results shown in Fig. 14 ▶ implies *n*_S_ ∼ ∊^0^. If *F*_b_ ∼ *n*_S_ *f*_Sb_, where *f*_Sb_ is the force at break per a single shish, the above results given by equations (5) and (6) infer also that *f*_Sb_ ∼ ∊^0^. This suggests that the final strength of the drawn fibres are controlled by the structure and properties of the SK developed in the as-spun fibres (*i.e. n*_S_ and *f*_Sb_) for the given hot-drawing conditions, so that the fibre spinning conditions are crucial to control the strength of the drawn fibres.

### 5.3. Universality and control parameters   

The evolution of the series of the dissipative structures found for the two experiments discussed in §4.2.1 and §4.2.2 is universal in the following sense. The same structural evolution into the SK was discovered to occur with time *t* after the step-up shear flow (§4.2.1) and with increasing distance *z* from the nozzle in the fibre spinning experiments (§4.2.2). The control parameter common to the two experiments may be the work done per unit volume *W* to the systems by the applied fields.

**Figure 17:**
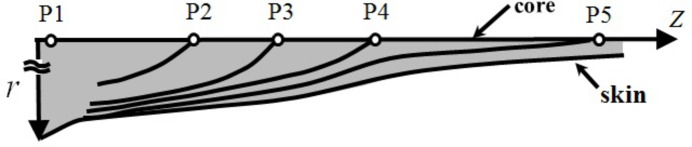
Skin–core structures of the as-spun fibres and the trajectories of the typical positions P2 to P5 in the parameter space of *z* and *r*, where the given dissipative structures 1 to 4 defined in Fig. 15 ▶ were formed by the given works *W* caused by the external field.

In the step-up shear experiments, *W* may be given by 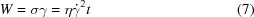 with the viscosity η given by (Bird *et al.*, 1977 ▶) 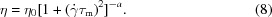
The necessary and sufficient conditions for the SK formation may be given respectively by 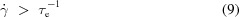
and 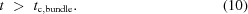
Although the formula for *W* as a function of *z* is still unknown in the case of the fibre spinning experiments, *W* is expected to certainly increase with *z*. Qualitatively, the increase of *z* corresponds to the increase of *t*, hence giving rise to the same structural evolution with *z* or *t* as discovered in the two experiments. In the case of the step-up shear, the larger the value η, 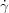 or σ is for a given set of other parameters, the faster is the structural evolution. In the case of the fibre spinning experiments also, the stress may be an important parameter for an intuitive understanding of the structural evolution as a function of *z* on the basis of *W*: the larger is the stress level imposed on the system, the smaller is the distance *z* at which the given dissipative structures develop. The skin–core structure of the as-spun fibre, as schematically shown in Fig. 17 ▶, will provide a support for this expectation.

The fibre running along the spinning line was cooled to room temperature, so that the fibre is subjected to a temperature gradient along its radial direction *r*, thereby *T* decreasing with *r*, which in turn gives rise to a stress gradient with *r*, thereby the stress increasing with *r*. Thus, the trajectories of the points P2 to P5 found along the centre (*r* = 0) of the spun fibre are expected to change with *r* as schematically shown in the Fig. 17 ▶. Upon increasing *r*, the given structure in the series of the structures evolved at P2 to P5 evolves at a shorter distance *z*, a qualitative trend which was experimentally confirmed.

### 5.4. Principles of pattern formation in open nonequilibrium systems as disclosed in this work   

**Figure 18:**
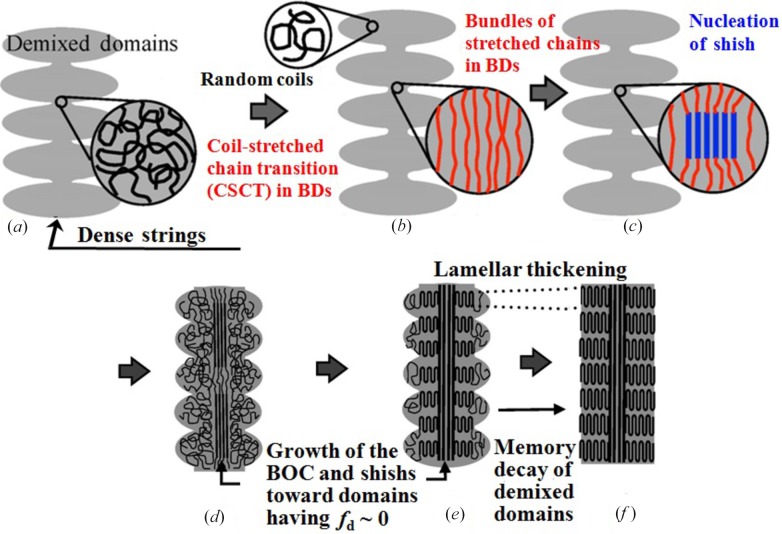
Principles concerning the unique pattern formation mechanism of SK in open nonequilibrium systems of polymer solutions. The pattern formation involves first formation of multi-length-scale, heterogeneous, amorphous dissipative structures, where the ordering into SK is triggered in local regions subjected to a large local stress concentration, followed by a series of the local events as illustrated in (*a*) to (*f*) even in the case when the bulk stress is relatively small. The local events (*a*) to (*d*) are also summarized in Table 2 ▶.

Here we would like to point out some principles of the pattern formation in the open nonequilibrium systems which were disclosed from the special crystallization mechanism of the SK as discussed in §4.2.2 (*e*). Fig. 18 ▶ and Table 2 ▶ summarize the local events (*a*) to (*c*) and their consequences given below, including the consequence (*d*), all of which are responsible for the special crystallization mechanism leading to the SK formation.

Local event (*a*). The dense strings composed of the demixed domains eventually formed the BDs between the domains with a small cross section area where chains are initially undeformed entangled coils, as illustrated in Fig. 18 ▶(*a*). As a consequence, in the BDs the local stress σ_l_ 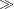 σ_b_, the average stress in the bulk. It is important to note that the self-assembly shown in Fig. 18 ▶(*a*) has a characteristic of a multi-length-scale, heterogeneous, amorphous precursor well suited for the special crystallization.

Local event (*b*). This consequence described above in turn causes the next local event of formation of the BOCs in the BDs *via* locally triggered CSCT as illustrated in the change from Figs. 18(*a*) to 18(*b*). As a consequence, the local and average orientational order parameters of chains as defined *f*_l_ and *f*_b_, respectively, the local and average melting points as defined *T*_ml_ and *T*_mb_, respectively, and the local and average supercooling as defined Δ*T*_ml_ and Δ*T*_mb_, respectively, satisfy the conditions shown for local event (*b*) in Table 2 ▶.

Local event (*c*). These consequences described above in local event (*b*) in turn cause the NG of shish to start from the BDs as illustrated in Fig. 18 ▶(*c*) and described in local event (*c*) in Table 2 ▶ at a very large rate. This event further brings about (*d*) growth of shishs extending to the domains having *f*_d_ ∼ 0, where *f*_d_ is the orientational order parameters of chains in the demixed domains, according to the autocatalytic orientations of relaxed chains in front of the growing shish crystal tips (Lieberwirth *et al.*, 2000 ▶), as shown in Fig. 18 ▶(*d*). Finally random coils in the demixed domains, which are coexisting with as-grown shishs, are epitaxically grown into the chain-folding kebab crystals from the surface of the shishs as schematically illustrated in the change from Fig. 18 ▶(*d*) to Fig. 18 ▶(*e*). These processes are primarily those responsible for the reduction of the extremely large conformational entropy of the systems compared with those for creating the other preceding amorphous precursors. The memory of the demixed domains will decay with *t* or *z* through the long-range chain diffusion within the SKs *via* the sliding chain diffusion within the crystals, as illustrated in the structure change from Fig. 18 ▶(*e*) to Fig. 18 ▶(*f*).

The unique ‘heterogeneous pattern formation’ mechanism unveiled for those particular open nonequilibrium systems studied in this work would not necessarily require the large bulk stress or field strength applied to systems. This is because the large local stress required for triggering the crystallization is effectively concentrated locally in the heterogeneous dissipative structure. We expect that the mechanism may be generally applicable to pattern formation in various open nonequilibrium systems.

## 6. Conclusions   

In this work, we elucidated the following principles for the pattern formation in open nonequilibrium systems composed of initially homogeneous polymer solutions.

(1) The cascading time evolution of the dissipative structures under external fields developed first amorphous and then crystalline textures in the self-assembling process of the SK, all of which cannot ever be created without the external fields.

(2) This is because nature most efficiently dissipates the energy imposed on systems by external fields *via* cascading reduction of large entropy and thereby large free energy barrier for ordering through the process (1) described above.

(3) In this work, the ECC were created *via* the formation of the SK first under the external fields and by the subsequent application of the multi-step hot-drawing to the SK, as another source of the external field. We elucidated the kinetic pathway from the homogeneous solution of random coils with maximum entropy to ECC with minimum entropy, through the cascading hierarchical structural evolution under the external fields.

(4) We elucidated the mechanisms and types of the dissipative structures formed in the cascading structural evolution process, although the free energy landscape D1 and D2 under the external fields are left unsolved as a future work.

(5) The sequential ordering under external fields obeys Ginzburg–Landau and Cahn–Hilliard laws: the large amorphous structures first evolve driven by the L–L phase separation under the low average stress σ_b_; the small structures such as the BOCs in BDs subsequently evolve, driven by the large stress σ_l_ locally concentrated. The crystallization of the polymers eventually occurred within the multi-length, heterogeneous, amorphous precursor evolved under the low bulk stress but the high local stress concentrated on the precursor. This special crystallization into the SK has the characteristic of the unique pattern formation mechanism as discussed in detail in §5.4 (see Fig. 18 ▶ and Table 2 ▶). The mechanism and process discovered here are anticipated to be applicable to the pattern formation in other open nonequilibrium systems.

## Figures and Tables

**Table 1:** Glossary of frequently used abbreviations

BD	Bridging domains	NG	Nucleation and growth
BOC	Bundle of oriented chains	ND	Neutral direction
CF	Concentration fluctuations	OM	Optical microscope
CSCT	Coin-to-stretched-chain transition	PLWCF	Plane wave concentration fluctuations
ECC	Extended-chain crystal texture	SALS	Small-angle light scattering
FD	Flow direction	SK	Shish-kebab crystalline texture
LL	Liquidliquid	UHMWPE	Ultrahigh molecular weight polyethylene
		UHMWaPS	Ultrahigh molecular weight atactic polystyrene

**Table 2:** Pattern formation mechanisms in the multi-length, heterogeneous, and amorphous precursor A unique feature found in the particular open non-equilibrium phenomenon involved in the SK formation. The local events (*a*) to (*c*) as well as the consequence (*d*) are indicated in Fig. 18 ▶ also.

Local events	Consequences
(*a*) Formation of the BDs within the dense strings	_l_ 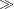 _b_, leading to the CSCT in the BDs
(*b*) Formation of the bundles of oriented chains (BOCs) in the BDs	*f*_l_ 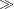 *f*_b_, *T*_ml_ 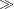 *T*_mb_, *T*_ml_ 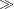 *T*_mb_
(*c*) The NG of shish starts from the BDs at a very large rate	(*d*) Growth of the BOCs and shishs into the domains having *f*_d_ 0
